# Comparing Ising and Spin Glass Dynamics in Financial Markets: A Complex Systems Approach to Asset Interdependence

**DOI:** 10.3390/e28030344

**Published:** 2026-03-19

**Authors:** Irina Georgescu, Jani Kinnunen

**Affiliations:** 1 Department of Economic Informatics and Cybernetics, Bucharest University of Economics, Calea Dorobanți, 15–7, Sector 1, 010552 Bucharest, Romania; 2 Department of Information Systems, Åbo Akademi University, Tuomiokirkontori 3, 20500 Turku, Finland; jani.kinnunen@abo.fi

**Keywords:** spin glass model, Ising model, financial networks, mutual information, systemic risk, entropy, commodity markets

## Abstract

This paper analyzes financial market interdependence from a statistical-physics perspective by comparing Ising and spin glass representations of asset interactions. Financial markets are modeled as complex systems in which collective behavior emerges from time-varying interaction structures. Using daily data for a diversified 15-asset commodity system, including precious metals, energy commodities, industrial metals and soft commodities, over the period 2020–2024, we construct rolling coupling matrices based on both linear correlations and nonlinear mutual information and embed them into Ising and Sherrington–Kirkpatrick-type interaction frameworks. While aggregate synchronization indicators—such as average coupling strength and the largest eigenvalue—exhibit similar dynamics across the two representations, the spin glass framework reveals substantially richer structural heterogeneity. Preserving the sign structure of the interactions leads to wider dispersion, higher variability and nontrivial network configurations that are suppressed in the Ising representation. The results identify the Ising model as a benchmark for market coherence. The spin glass model is essential for capturing heterogeneous interactions and nonlinear dependence in financial markets.

## 1. Introduction

Financial markets exhibit many features typical of complex adaptive systems, including nonlinear interactions, collective behavior, and abrupt regime shifts. Empirical regularities such as volatility clustering, contagion, and market crashes suggest that market dynamics cannot be fully understood through representative-agent or equilibrium-based models alone [1]. Instead, these phenomena emerge from interactions among heterogeneous agents and assets, motivating the use of statistical physics approaches in finance ([2,3,4]). Among the models adopted in econophysics and sociophysics, the Ising model occupies a central position due to its simplicity and ability to capture collective alignment and phase transitions. Originally introduced to study ferromagnetism ([5,6]), the Ising framework has been widely applied to social and economic systems, including opinion dynamics, decision-making, and financial markets, where binary variables naturally represent buy–sell decisions or bullish–bearish sentiment ([7,8,9]). In financial contexts, Ising-type interactions have been shown to generate herding behavior, market synchronization, and crash-like dynamics, especially in highly correlated environments ([10,11]).

However, real financial markets are characterized by heterogeneous and competing interactions, which cannot be fully captured by purely ferromagnetic models. In this context, the spin glass model, originally developed to describe disordered magnetic systems with frustrated interactions ([12,13]), provides a natural extension. Spin glass dynamics are associated with rugged interaction landscapes, multiple metastable states, and persistent non-equilibrium behavior. These features bear qualitative resemblance to financial markets during turbulent periods ([2,14]). Galam [8] and Mullick & Sen [15] highlight that Ising-like collective alignment may coexist with spin-glass-type disorder in socio-economic systems. Comparative analysis between these frameworks is relevant for understanding market coherence and systemic instability.

Motivated by this perspective, the present study compares Ising and spin glass representations of financial market interactions. The focus is on how different assumptions regarding coupling structure influence measures of market coherence, heterogeneity and synchronization. We embed empirically estimated interaction matrices into Ising and spin glass-type formulations as analytical frameworks for organizing and interpreting market interdependence. This approach allows us to assess when simplified Ising-type representations are an appropriate benchmark when more general spin-glass formulations are required to capture heterogeneous and nonlinear interactions in financial markets.

The paper is structured as follows. Section 2 introduces the theoretical framework underpinning the analysis, presenting the Ising and spin glass models and their interpretation in the context of financial market interdependence. Section 3 reviews the related literature. Section 4 reports the empirical results based on a diversified system of commodity markets, comparing linear and nonlinear dependence structures under the two modeling frameworks. Section 5 concludes by summarizing the main findings, discussing their implications for systemic risk and portfolio diversification, and outlining directions for future research. Additional robustness checks are reported in the appendices. Appendix A presents the Diebold–Yilmaz connectedness analysis used as an external benchmark for synchronization indicators. Appendix B examines the sensitivity of the results to the rolling window length and mutual information estimation. Appendix C introduces a simple dynamic extension based on Glauber dynamics to illustrate the behavior of the interaction system.

## 2. Theoretical Framework

In recent decades, the conceptual and mathematical tools of statistical physics have been increasingly adopted in the analysis of financial markets and macroeconomic systems ([2,3]). This interdisciplinary approach seeks to understand collective market behavior by drawing analogies with interacting many-body systems.

Financial systems can be regarded as complex adaptive systems, consisting of many interacting components (e.g., traders, institutions, or assets) whose collective behavior gives rise to emergent phenomena such as volatility clustering, market synchronization, contagion and crises ([4,16,17]).

In this analogy, statistical dependencies between financial assets can be interpreted as interactions analogous to spin couplings in magnetic systems. Statistical physics models such as the Ising and spin glass frameworks provide representations for studying how local interaction patterns (asset correlations) aggregate into indicators of market coherence or heterogeneity (market regimes) [18].

### 2.1. The Ising Model

The Ising model, originally proposed by Lenz (1920) [6] and developed by Ising (1925) [5] to explain ferromagnetism through pairwise interactions among binary spins, has become a canonical framework for studying collective behavior in interacting systems.

The Ising framework has been extended to model collective dynamics in economics, opinion formation, and markets ([7,9]). In this study, the Ising framework is not used as a physical model but as a benchmark for summarizing empirical dependence structures in financial markets. We adopt an Ising-type representation in which non-negative interaction strengths are constructed from the absolute values of empirical dependence measures between asset returns. This representation captures the degree of collective alignment and market-wide synchronization implied by asset co-movements.

### 2.2. The Spin Glass Model

The spin glass model generalizes the Ising system by allowing heterogeneous and frustrated couplings, i.e., interactions that can be both positive and negative ([12,13]).

In the original Sherrington–Kirkpatrick formulation, interaction strengths are modeled as random variables with zero mean and finite variance. In this study, however, interaction strengths are empirically estimated from financial data, resulting in a heterogeneous dependence structure that preserves both the magnitude and sign of empirical co-movements between assets.

From a financial perspective, this heterogeneity reflects conflicting relationships among assets, sectors, or instruments that sometimes move together (positive dependence) and sometimes diverge (negative dependence). Unlike the Ising benchmark, which suppresses sign heterogeneity by construction, the spin-glass representation retains this information, allowing for direct measurement of frustration and structural disorder in financial networks.

The resulting interaction structure is characterized by competing dependencies and the absence of a single dominant alignment pattern, which is consistent with the multistable and heterogeneous nature of real financial markets, particularly during periods of stress, regime transitions, and elevated systemic risk ([3,14]).

In the empirical analysis, the coupling matrix J is constructed directly from the correlation matrix of asset log returns. For the Ising benchmark, couplings are defined as the absolute value of correlations, capturing the degree of collective alignment. For the spin-glass representation, signed correlations are retained, preserving interaction heterogeneity and frustration. In all cases, diagonal elements are set to zero to exclude self-interactions.

Table 1 compares Ising and spin glass models.

The Ising model captures macroscopic coordination—such as bubbles, panic, or synchronized trading—while the spin glass describes the conflict and heterogeneity of real financial systems.

This interpretation is consistent with recent theoretical models of bubbles that characterize them as intrinsically unstable and inefficient regimes, driven by endogenous market failures and limits to arbitrage [19]. In our framework, such unstable regimes are represented by Ising-like configurations, where excessive alignment and reduced heterogeneity amplify systemic vulnerability.

Empirically, financial markets often oscillate between Ising-like (highly coordinated) and spin-glass-like (heterogeneous and disordered) regimes, particularly during transitions from stability to crisis ([10,11]).

The algorithm for constructing spin glass and Ising market representations follows the procedure:

Step 1 For each commodity *i*, daily log returns are computed as $r_{i}\left(t\right)=\ln\left(P_{i}\left(t\right)\right)-ln(P_{i}(t-1))$, where $P_{i}\left(t\right)$ is the price of commodity *i* at time *t*.

Step 2 For a window of length w=90 trading days, we compute:

$C_{ij}\left(t\right)=corr(r_{i}\left(t-w+1:t\right),r_{j}\left(t-w+1:t\right))$. The diagonal is set to zero: $C_{ii}\left(t\right)=0$.

This produces a time-varying interaction matrix.

Step 3 The spin-glass interaction matrix preserves the sign of correlations:

$J_{ij}^{SG}=C_{ij}\left(t\right)$. Positive values represent cooperative interactions, while negative values represent frustration or competition.

Step 4 The Ising representation focuses on the strength of interactions regardless of sign: $J_{ij}^{Ising}={|C}_{ij}\left(t\right)|$.

Step 5 Each matrix is interpreted as a weighted network where nodes are commodities and edges correspond to the interaction strength $J_{ij}(t)$.

### 2.3. Mutual Information as a Nonlinear Dependence Measure

Traditional correlation captures only linear dependence between financial assets. However, financial markets are characterized by nonlinear interactions, asymmetric responses, and tail co-movements during stress periods. Mutual Information (*MI*) offers a more general and robust measure of statistical dependence. It detects both linear and nonlinear relationships without requiring distributional assumptions ([20,21]).

For two discretized return series *X* and *Y*, *MI* is defined as:

(1) $$MI\left(X,Y\right)=\sum_{x}\sum_{y}p\left(x,y\right)log\frac{p(x,y)}{p\left(x\right)p(y)}$$

In Formula (1), p(x,y) denotes the joint probability function of the discrete variables *X* and *Y*. p(x) and p(y) are their marginal distributions. The indices *x* and *y* label the discrete states obtained after discretization. In the empirical implementation, each return series is discretized into four quantile-based bins (quartiles), ensuring equal probability mass in each bin. We refer to these as quantile-based bins with four categories.

In principle, *MI* can be defined for continuous variables using a formulation based on probability density functions:

(2) $$MI\left(X,Y\right)=\iint f_{XY}\left(x,y\right)log\frac{f_{XY}(x,y)}{f_{X}(x,y)f_{Y}(x,y)}dxdy$$

In empirical applications the joint density is unknown and must be estimated from finite samples. Therefore, we adopt a discretized approximation based on empirical probability masses. The summation form in Equation (1) corresponds to the estimator after discretization of returns into bins.

Li et al. [22] note that discretizing continuous data into common bins is a common strategy to approximate complex distributions. Such approximations may fail to capture sharp changes in density unless a large number of bins is used. Discretization introduces a bias-variance trade-off. A small number of bins reduces variance but may underestimate dependence due to a coarse approximation. Too many bins increase estimation noise in finite samples. Quantile-based discretization has theoretical support in the information-theory literature. For example, Gupta et al. [23] propose a quantile-spacing method that partitions the data into equal-probability intervals, showing that such partitions can produce accurate entropy estimates without requiring tuning of bin widths.

In our empirical analysis, discretization is performed using quantile-based binning implemented in R Studio 2023.12.1 Build 402. Robustness checks confirm that alternative binning schemes (e.g., terciles, adaptive bins) yield qualitatively similar results.

MI-based networks differ structurally from correlation networks in several aspects. First, MI is non-negative by construction and quantifies the strength of statistical dependence independently of its sign [20]. Secondly, MI assigns larger weights to tail-dependence, which is important in commodity and energy markets where price spikes and stress episodes dominate information transmission [24]. Thirdly, MI captures nonlinear synchronization patterns that linear correlation matrices cannot detect [25].

In the spin glass analogy, MI can be interpreted as defining an alternative dependence-based coupling matrix $J^{MI}$, complementing the correlation-based matrix $J^{corr}$. By capturing the total dependence, MI uncovers hidden risk channels, nonlinear contagion pathways, and early-warning signatures of systemic crises [25,26]. It provides a more comprehensive representation of financial market interactions when combined with the spin glass framework.

### 2.4. Structural Entropy Measures of Market Dependence

Entropy-based diagnostics provide a natural and well-established framework to quantify structural uncertainty, nonlinear dependence, and interaction complexity in financial markets. Different entropy measures capture distinct aspects of coupling heterogeneity. In our empirical implementation, three entropy measures are considered.

(a) Shannon Entropy of Couplings

(3) $$H_{S}=-\sum_{i<j}p_{ij}log\;p_{ij}\;\mathrm{with}\;p_{ij}=\frac{\left|J_{ij}\right|}{\sum_{i<j}\left|J_{ij}\right|}$$

In the empirical implementation, entropy is computed from normalized absolute coupling strengths, making it insensitive to the sign of interactions. The interaction matrix $J_{ij}$ describes the empirical dependence structure of the system. Each element represents the coupling strength between assets i and j. The normalization (3) transforms the absolute interaction strengths into a discrete probability distribution over all distinct interacting pairs in the network. The Shannon entropy $H_{S}$ provides a global indicator of structural disorder. A higher value of $H_{S}$ reflects a more heterogeneous and evenly distributed coupling structure. Lower entropy indicates a more concentrated structure dominated by a small number of strong dependencies. This entropy measure is therefore informative about the degree of systemic organization and the distribution of interaction intensities in the financial network.

(b) Rényi Entropy

Given the tendency of financial networks to exhibit uneven clustering and concentrated risk transmission, Rényi entropy offers a powerful tool for characterizing the heterogeneity in coupling structures. Rényi entropy of order α is defined as:

(4) $$H_{R}\left(\alpha \right)=\frac{1}{1-\alpha }log\sum_{i<j}p_{ij}^{\alpha }$$

where $p_{ij}$ denotes the normalized interaction strengths defined in Equation (3).

Because the exponent *α* amplifies the contribution of larger couplings, Rényi entropy places greater weight on strong systemic interactions. $H_{R}\left(\alpha \right)$ is informative for assessing the degree of coupling concentration [27]. Lower values of $H_{R}\left(\alpha \right)$ reveal increasing concentration and the formation of dominant systemic clusters. Higher values indicate a more homogeneous, less concentrated dependence structure in the financial network. In the empirical analysis, we set *α* = 2, which emphasizes dominant couplings and highlights the concentration of systemic dependence.

(c) Tsallis Entropy

To capture the nonlinear dependence structure, long-range interactions, and heavy-tailed behavior observed in financial markets, we include the Tsallis entropy measure in our analysis [28]. Tsallis entropy introduces the entropic index *q*, which controls the sensitivity of the measure to rare and extreme events. When *q* = 1, the Tsallis entropy reduces to the classical Shannon entropy. For *q* > 1, the measure assigns increasing weight to large and infrequent interaction strengths, making it suitable for financial systems characterized by heavy tails, volatility clustering, and strong coupling during turbulent periods. Values of *q* < 1 emphasize frequent, small-impact interactions. Tsallis entropy of order q is defined as:

(5) $$H_{T}\left(q\right)=\frac{1-\sum_{i<j}p_{ij}^{q}}{q-1}$$

where $p_{ij}$ denotes the normalized coupling strengths defined in Equation (3).

This formulation allows multi-scale dependence patterns and interaction heterogeneity, enabling a more realistic characterization of complexity in financial networks. Higher values of $H_{T}\left(q\right)$ indicate broader and more intricate coupling structures.

In the empirical interpretation, we fixed the entropic index *q* = 2, which enhances the contribution of strong interactions and tail dependence while remaining robust to noise [28].

### 2.5. Rolling Coupling Matrices and Dynamic Dependence Regimes

Financial markets exhibit pronounced temporal variability. The strength and structure of asset interdependencies evolve continuously in response to changing economic conditions. A natural way to capture this evolution is through rolling-window coupling matrices, where the correlation-based or mutual-information-based coupling matrix J(t) is recomputed over a moving time window w. This procedure yields a sequence of time-varying interaction matrices that describe the evolving dependence structure of the market. In the empirical analysis, rolling correlation matrices are used for linear benchmarking and spectral indicators. Rolling mutual-information matrices are employed to capture nonlinear dependence.

We denote by $J^{MI}(t)$ the rolling mutual-information-based coupling matrix. Changes in $J^{MI}(t)$ reflect the reorganization of pairwise asset dependencies and reveal how local interactions aggregate into distinct global market regimes over time.

This dynamic perspective is central to the econophysics literature, where shifts in the correlation structure are known to precede and accompany market crises ([3,10,11]). Sudden increases in co-movement signify systemic fragility ([29,30]).

We can identify regime shifts and abrupt reorganizations in market dependence structure. These transitions correspond to changes from highly synchronized to more heterogeneous configurations of asset interactions, in analogy with ordered and disordered states in complex systems [2,12]. Periods of strong synchronization are associated with co-movement and collective behavior, observed during contagion [31], herding [32], and crisis propagation [33]. Less synchronized regimes reflect fragmented interaction structures ([34,35]). Changes in the coupling matrix are interpreted as structural reorganizations of the dependence network. Market regimes can be described as stable, transitional or crisis-driven ([36,37]).

This interpretation is in line with empirical evidence showing that financial correlations increase under stress, a stylized fact documented across equities, commodities, and multi-asset portfolios ([29,38]).

### 2.6. Largest Eigenvalue Dynamics $\lambda_{1}(t)$

The largest eigenvalue of the rolling coupling matrix, $\lambda_{1}\left(t\right)=\max eig\;J(t)$ provides a system-level indicator of collective market behavior. In correlation-based representations of financial markets, the dominant eigenvalue captures the strength of the market mode—the common component that drives synchronized movements across assets ([10,39]). When $\lambda_{1}(t)$ rises sharply, the market enters a regime of heightened co-movement, reflecting strong alignment of asset returns and an erosion of diversification advantages. Such episodes coincide with stress periods, contagion, and systemic fragility, during which local shocks propagate rapidly through the network of financial linkages. Conversely, lower values of $\lambda_{1}\left(t\right)$ signal a more fragmented or heterogeneous market structure in which idiosyncratic factors dominate and interdependencies weaken. Tracking the temporal evolution of $\lambda_{1}\left(t\right)$ therefore offers a powerful early-warning indicator. Sustained increases or abrupt spikes may foreshadow transitions toward crisis regimes [40]. Within the spin-glass-inspired interpretation adopted in this study, fluctuations in $\lambda_{1}\left(t\right)$ are understood as reflecting structural reorganizations of the dependence networks.

### 2.7. Parallel Financial Networks (Returns Layer and Volatility Layer)

To compare different channels of dependence in financial markets, we construct two separate network layers using the same set of N assets. The first layer is a return-based coupling matrix, $J^{\left(ret\right)}\left(t\right)=\{J_{ij}^{\left(ret\right)}\left(t\right)\}$, computed from rolling-window log return correlations. This layer captures fast market adjustments, short-term co-movement, and immediate responses to news and shocks. The second layer is a volatility-based coupling matrix, $J^{\left(vol\right)}\left(t\right)=\{J_{ij}^{\left(vol\right)}\left(t\right)\}$, constructed from rolling-window correlations of realized volatilities. Because volatility evolves more slowly and exhibits persistence, this layer reflects structural risk pressures, such as volatility clustering, and the gradual buildup of systemic stress.

In the empirical analysis, these two layers are treated as parallel but independent network representations. Their comparison highlights important differences in market dynamics. The return layer fluctuates rapidly, exhibiting bursts of synchronization around major news events. The volatility layer evolves more gradually and captures persistent stress conditions.

### 2.8. Statistical Comparison of Regimes

To evaluate whether the Ising and spin glass formulations capture distinct dependence structures, we compare statistical properties of their coupling matrices. For each rolling window, the interaction strength is summarized through the average coupling, defined as

(6) $$\langle J(t)\rangle =\frac{1}{N(N-1)}\sum_{i\neq j}J_{it}(t)$$

computed as the mean of the off-diagonal entries of the coupling matrix. This measure provides a compact scalar indicator of market organization and has been used in financial literature to characterize changes in market coherence [37]. Using this definition, we compare the distributions of $\langle J(t)\rangle$ across the Ising and spin-glass-type coupling constructions, together with the corresponding distributions of the largest eigenvalue $\lambda_{1}(t)$, which reflects the strength of the dominant market mode [2,3]. Distributional differences are assessed using the Kolmogorov–Smirnov test, while mean differences are evaluated through Welch’s *t*-test. Effect sizes are quantified using Cohen’s d [41]. Significant differences in $\langle J(t)\rangle$ indicate that the models encode different degrees of clustering and coherence. Similar $\lambda_{1}(t)$ values suggest that both representations may remain influenced by a common market factor. This multi-statistic comparison aligns with systemic-risk methodologies that differentiate market regimes using structural and spectral diagnostics [37].

From an economic perspective, higher average couplings $\langle J(t)\rangle$ under the Ising-type construction correspond to periods of stronger collective alignment and herding, consistent with episodes of market-wide synchronization ([2,3]). The spin-glass-type construction, by preserving sign heterogeneity in interactions, captures more fragmented and competitive dependence patterns ([2,10,11]).

Mutual-information-based couplings enhance this comparison by revealing nonlinear dependencies that correlation matrices alone cannot detect [42,43]. Time-varying spectral indicators, such as $\lambda_{1}(t)$ and entropy-based measures enhance sensitivity to regime transitions [37]. The comparison between return-based and volatility-based networks illustrates how short-term co-movement differs from slower dependence associated with volatility persistence and risk clustering.

## 3. Literature Review

Statistical physics has long provided a powerful framework for studying collective phenomena in systems composed of many interacting units. Within this tradition, Ising and spin glass models have become central tools for analyzing complex socio-economic and financial systems, where macroscopic patterns emerge from heterogeneous and interacting components [2,3]). Mullick & Sen [15] confirm that Ising-inspired models remain foundational for modeling collective dynamics in sociophysics, finance, and networked systems, particularly in contexts characterized by abrupt regime changes.

The Ising model, originally introduced to describe ferromagnetism (Ising [5]), has been adapted to represent binary decision-making and coordination processes. In economic and financial applications, Ising-type frameworks are commonly used to model alignment, herding, and synchronization effects, where increasing interaction strength leads to coherent collective behavior. Empirical motivation for such models is provided by evidence that asset correlations intensify during turbulent periods, amplifying systemic risk [10,11]. Glauber’s kinetic Ising model [44] provides stochastic single-spin–flip dynamics that satisfy detailed balance, allowing the study of relaxation processes and nonequilibrium evolution toward equilibrium in interacting spin systems. Campajola et al. [45] extended Ising modeling toward kinetic and data-driven formulations, emphasizing time-varying interactions and nonequilibrium dynamics as essential features of real-world systems.

Giorgio [46] has explored the ability of Ising-type models to reproduce key stylized facts of financial markets, including volatility clustering and heavy-tailed return distributions, reinforcing the relevance of phase-transition analogies in financial dynamics. Cividino et al. [47] propose an agent-based model with Ising-inspired imitation dynamics generalized to a multi-asset O(n) framework, showing how herding-driven interactions reproduce stylized facts and generate bubble-like regime transitions in financial markets. Mullick and Sen [15] emphasize the importance of evidence-driven calibration and the choice of dynamical update rules (e.g., Glauber-type versus non-equilibrium dynamics) in moving from qualitative analogies toward empirically testable Ising-like models.

Sakuler et al. [48] prove that Ising-type formulations combined with annealing dynamics can be calibrated and tested on real financial data, yielding practical portfolio optimization results.

In contrast, spin glass models explicitly incorporate heterogeneity and competing interactions through random and signed couplings, giving rise to frustration and rugged energy landscapes with multiple metastable states [12,13]. These features make spin glasses particularly suitable for systems where coherent alignment is incomplete or unstable. Marsili [36] highlights their relevance for modeling complex dependence structures in financial markets and social systems, where regimes shift and multiple quasi-stable configurations coexist. Korbel et al. [49] show that spin-glass-inspired self-assembly mechanisms can reproduce realistic group-size distributions by balancing homophily-driven alignment and competing interactions. [18,50] argue that economic systems are complex and heterogeneous, so their aggregate behavior cannot be understood using representative-agent models. Drawing on spin-glass theory, they show that interactions, feedbacks, and nonlinearities naturally lead to multiple equilibria, instability, and coordination failures in economic and financial systems.

A recent trend is the integration of spin-based models with network and information-theoretic approaches. Nonlinear dependence measures such as mutual information have been used to construct interaction networks that capture relationships beyond linear correlation [20,25]. Systemic-risk research emphasizes how network topology shapes fragility and contagion [51,52]. Georgescu and Kinnunen [53] apply entropy- and chaos-based methods to commodity markets and show that mutual information effectively captures nonlinear and time-varying dependencies that are not detectable using linear correlation measures. Georgescu and Kinnunen [54] apply a spin-glass framework combined with a multiple-threshold nonlinear ARDL model to show that market volatility affects systemic financial disorder in a regime-dependent and asymmetric manner. These developments position Ising and spin glass models as complementary tools for studying collective order, heterogeneity, and regime transitions in complex systems.

Kirman-type herding models represent another influential class of binary-state models. Originating from Kirmans’s [55] ant recruitment model, these approaches describe agents switching between alternative states under the influence of individual preferences and social imitations. Alfarano et al. [56] extend Kirman’s ant recruitment process to financial markets by modeling interactions between chartists and fundamentalists, showing that the resulting herding dynamics can generate bubbles. Kononovicius and Gontis [57] introduce a variable event time scale that reflects changing trading activity, showing that the resulting stochastic dynamics reproduce long-range memory and power-law statistics observed in financial markets. The model by Vilela et al. [58] introduces heterogeneous agents—contrarians and noise traders—whose local and global interactions generate stylized facts of financial markets, including volatility clustering, power-law return distributions, and long-range correlations in absolute returns.

## 4. Empirical Results

This section presents the empirical findings obtained from applying the proposed interaction-based framework to a diversified set of 15 major commodity assets, covering several commodity classes, over the period 1 January 2020 to 31 December 2024. The dataset includes precious metals (Gold, Silver, Platinum, Palladium), energy commodities (WTI Crude Oil, Brent Crude Oil, Natural Gas, Heating Oil), an industrial metal (Copper), agricultural grains (Wheat, Corn, Soybeans), and soft commodities (Coffee, Sugar, Cotton). Daily closing prices were extracted from *Yahoo Finance*, and log returns were computed to ensure comparability across assets. The analysis focuses on uncovering the structural properties of market dependence patterns through both linear and nonlinear measures. Rolling-window correlation matrices are employed to capture time-varying linear co-movement. Mutual-information-based couplings are used to identify nonlinear and tail-dependent relationships.

**Figure 1:**
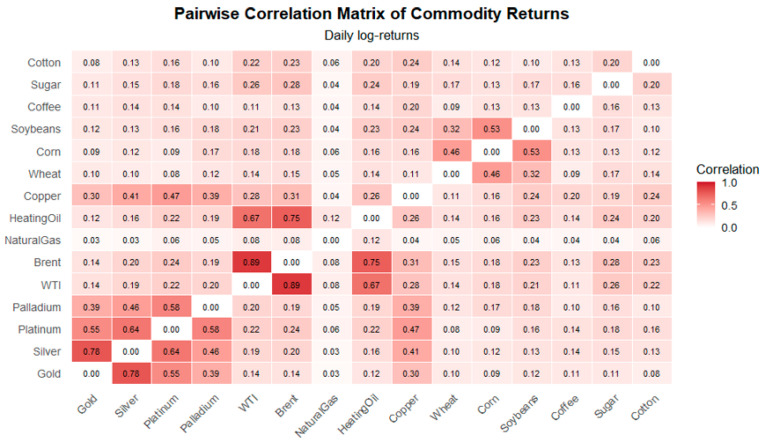
Pairwise correlation heatmap of daily commodity log returns (2020–2024).

The heatmap in Figure 1 reports Pearson correlation coefficients between daily log returns for 15 commodity futures. Diagonal elements are set to zero to emphasize cross-asset interactions. First, strong intra-sectoral synchronization is obvious among precious metals. Gold and Silver exhibit the highest correlation (ρ ≈ 0.78), followed by Platinum and Palladium, reflecting their shared exposure to macroeconomic uncertainty, inflation hedging, and financial market sentiment. Energy commodities show pronounced co-movement, with WTI and Brent crude displaying a very high correlation (ρ ≈ 0.89), consistent with their substitutability and common global demand–supply drivers. Second, cross-sector correlations are generally moderate. Industrial metals such as Copper exhibit moderate correlations with both energy and precious metals. Natural Gas displays weak correlations with most other commodities. Third, agricultural commodities exhibit heterogeneous dependence structures. Grains (Wheat, Corn, Soybeans) are moderately correlated among themselves, while soft commodities (Coffee, Sugar, Cotton) show weaker and more fragmented relationships.

The correlation matrix reveals a highly heterogeneous interaction structure, characterized by strong clustering within commodity groups and weaker inter-group linkages. This empirical pattern motivates the use of spin-glass representations. At the same time, because correlations are predominantly positive, the static correlation structure provides a natural Ising-type benchmark capturing aggregate market synchronization. The distinction between the Ising and spin-glass frameworks does not arise at the level of static correlations.

**Figure 2:**
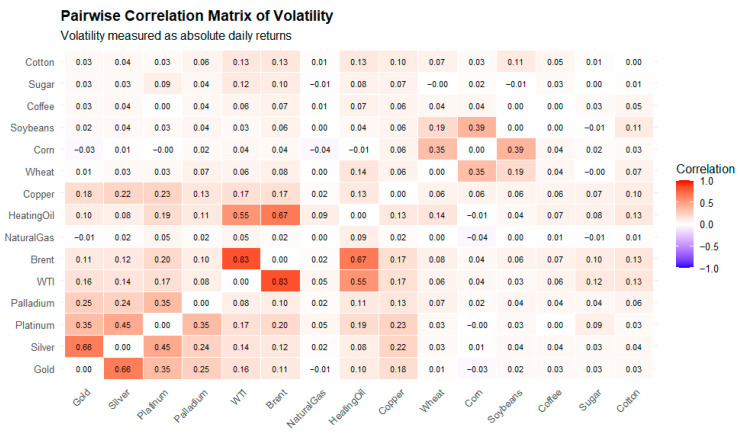
Pairwise correlation matrix of commodity volatility.

The heatmap in Figure 2 displays the cross-asset dependence structure of volatility across the 15 commodities. Volatility is measured as absolute daily log returns. Strong positive correlations are observed within energy markets, particularly between WTI and Brent crude, indicating synchronized risk dynamics driven by common supply, demand, and geopolitical shocks [59]. Precious metals, especially Gold and Silver, also exhibit notable volatility co-movement. Natural Gas volatility remains weakly correlated with most other commodities. Agricultural commodities show low to moderate volatility correlations, suggesting more segmented and product-specific risk transmission mechanisms. It follows that systemic risk in commodity markets is highly sector-dependent.

Table 2 reports Shannon, Rényi, and Tsallis entropy values computed from the empirical dependence structure of the commodity market. All three entropy measures take identical values under the Ising and spin glass representations. This result reflects a specific empirical characteristic of the data rather than a methodological limitation. The equality of entropy values arises because the empirical correlation structure is dominated by positive co-movements. Since entropy measures the dispersion of interaction strengths rather than their sign, both the Ising and spin glass representations encode the same level of structural heterogeneity, resulting in identical entropy values. During the analyzed period, 2020–2024, market organization has been dominated by collective synchronization rather than frustration or conflict, leading to identical entropy outcomes. However, this outcome may depend on the specific market environment considered. In other financial contexts where negative correlations are more prevalent, the Ising and spin-glass representations may lead to different entropy values. The analysis can be extended to additional asset classes or market regimes. Entropy measures can be defined on signed or nonlinear dependence networks, which may reveal structural differences not captured by correlation-based measures. Fiedor [25] showed that nonlinear dependence measures can reveal interactions that differ from standard correlation approaches. Zhao et al. [60] use a causal network model based on transfer entropy and show that the structure of information flows among commodities changes significantly during major crises such as economic shocks and the Russia–Ukraine conflict.

The raw entropy values reported in Table 2 should be interpreted as indicators of dispersion of interaction strengths, rather than as measures of directional structure. Entropy depends on the distribution of coupling magnitudes across the network and is insensitive to the sign of correlations. Because the empirical commodity correlation matrix is dominated by positive relationships, both the Ising and spin-glass constructions generate similar distributions. Therefore, the entropy measures coincide. The differences between the models emerge in the signed interaction topology.

**Figure 3:**
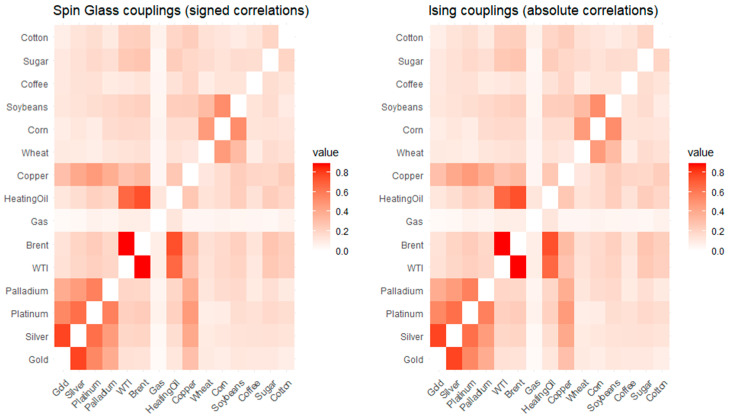
Spin glass and Ising coupling heatmaps.

Figure 3 shows that the spin glass (signed) and Ising (absolute) coupling heatmaps are visually indistinguishable, reflecting the dominance of positive correlations and the negligible role of interaction sign in the empirical dependence structure.

**Figure 4:**
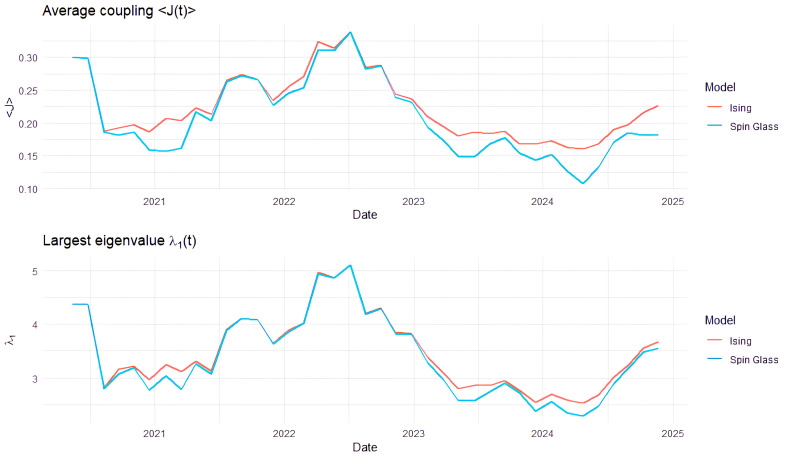
Time-varying average coupling and largest eigenvalue under Ising and spin glass representations.

Figure 4 illustrates the time evolution of the average coupling $\langle J(t)\rangle$ and the largest eigenvalue $\lambda_{1}(t)$ for the Ising and spin glass representation of commodity return dependencies. One can see that market synchronization is not uniformly elevated during 2020–2021, despite the COVID-19 shock. Both $\langle J(t)\rangle$ and $\lambda_{1}(t)$ remain moderate during the early pandemic period and increase more significantly during 2022, followed by a decline and partial recovery after that. It follows that the COVID-19 crisis was characterized by heterogeneous responses in commodity markets. Even if financial stress was high, different commodities reacted differently. Energy markets experienced demand declines and later rebounds. Precious metals reacted as safe havens. Agricultural commodities followed more idiosyncratic supply-demand dynamics. As a result, cross-asset co-movement remained limited.

The pronounced peak around 2022 coincides with a period of geopolitical and macroeconomic shocks, including the Russia–Ukraine war, energy supply disruptions and inflationary pressures. Several commodity markets have been affected at the same time, leading to stronger alignment across assets and higher average coupling.

The subsequent decline in both indicators during 2023–2024 suggests a re-fragmentation of market dynamics. Supply chains adjusted, and policy uncertainty evolved. The near-identical Ising and spin-glass curves indicate that both models convey the same synchronization trend.

Although empirical correlations are predominantly positive, the Ising and spin-glass coupling matrices are not strictly identical at each rolling window. Small and intermittent negative correlations are preserved in the spin-glass construction and removed in the Ising benchmark through absolute-value transformation. When aggregated into average coupling and spectral indicators, these local differences accumulate and produce systematic divergences in $\langle J(t)\rangle$ and $\lambda_{1}(t)$. The near-overlap of the curves therefore reflects a largely synchronized market regime, while the remaining gap quantifies the contribution of interaction heterogeneity suppressed by the Ising representation.

**Figure 5:**
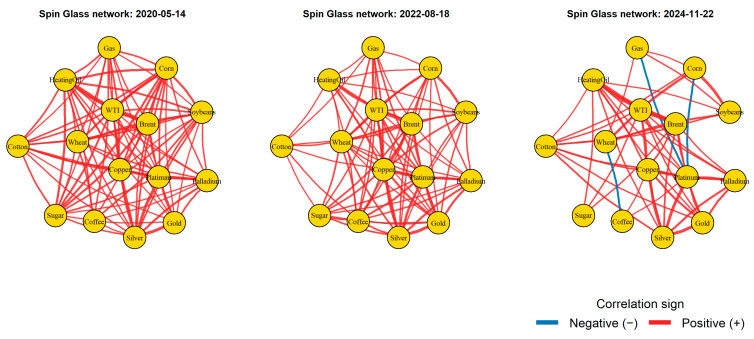
Evolution of the spin-glass interaction network across market regimes. Note: Edge colors indicate the sign of pairwise correlations (blue for negative and red for positive), while edge thickness is proportional to the absolute strength of the interaction. Only correlations with absolute value greater than 0.15 are displayed for clarity.

The similarity between the Ising and spin-glass indicators should not be interpreted as evidence that the two representations are identical. The difference consists of how interaction signs are treated. The Ising construction removes the sign of the correlation and therefore describes the intensity of market synchronization. The spin-glass formulation preserves sign interactions and retains information about sectoral fragmentation. Even if negative correlations are infrequent in this chosen set of commodities, they appear intermittently across rolling windows and generate locally structural differences in the interaction network. These differences become visible in Figure 5, where the spin-glass model reveals the coexistence of cooperative and antagonistic relationships that are suppressed in the Ising model.

Figure 5 displays the spin-glass networks constructed from rolling matrices at three representative dates (14 May 2020, 18 August 2022, and 22 November 2024), selected from the rolling-window analysis to illustrate different market conditions. Nodes represent commodities, while edges represent pairwise interactions derived from signed correlations. Edge thickness reflects interaction strength. Red edges indicate positive correlations, while blue edges indicate negative correlations. 

In the left panel (14 May 2020), the network is dominated by positive links and relatively homogeneous connectivity. It indicates a synchronized market structure during the early post-shock phase of the COVID-19 period. Most commodities move in the same direction, reflecting common global demand and liquidity effects.

The middle panel (18 August 2022) shows stronger and denser positive interactions, particularly among energy and industrial commodities. It reflects heightened synchronization during the energy and commodity price shocks associated with the Russia–Ukraine conflict, when geopolitical stress amplified co-movements across markets.

In the right panel (22 November 2024), the network becomes more heterogeneous, with the appearance of several negative (blue) links alongside positive ones. This indicates increasing fragmentation across commodity groups, consistent with a transition toward a more disordered spin-glass-like regime in which diversification reoccurs and market coordination weakens.

One can conclude from Figure 5 how the commodity market interaction structure evolves in time from ordered and synchronized states toward more heterogeneous and frustrated configurations. The changing balance between positive and negative couplings highlights the usefulness of the spin-glass framework for capturing regime shifts, structural reorganization, and the buildup or dissipation of systemic dependence in financial markets.

The evolution of edge colors across the three selected dates reflects changes in the correlation structure of the commodity market. On 14 May 2020, most edges are red, indicating that correlations are mainly positive and commodities tend to move together. By 18 August 2022, the network remains strongly connected, with several intense positive links. By 22 November 2024, several edges turn blue, indicating the presence of some negative correlations. The market evolves from a more ordered state to a more fragmented structure.

The spin-glass representation captures richer structural heterogeneity because it preserves the signed interaction structure of the market. Positive couplings represent synchronized price movements, while negative ones correspond to sectoral diversification. When correlations are transformed into absolute values in the Ising model, this information disappears. The system is reduced to a configuration in which interactions promote alignment. This interpretation is consistent with Lisewski and Lichtarge [61], who prove that financial systems exhibit both negative and positive correlations and can be viewed as a spin-glass model.

**Figure 6:**
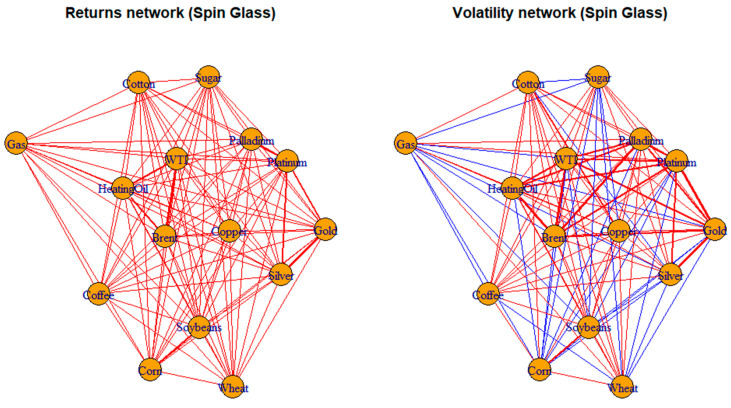
Spin glass networks based on returns and volatility. Note: Edge colors indicate the sign of correlations: red denotes positive correlations, while blue denotes negative correlations; line thickness reflects interaction strength.

Figure 6 compares spin-glass interaction networks constructed from return-based correlations (left panel) and volatility-based correlations (right panel). Nodes represent commodities. Edges reflect signed interaction strengths: red links indicate positive dependence, and blue links indicate negative dependence. Edge thickness is proportional to interaction magnitude.

The returns-based network (left panel) is dominated by positive interactions, indicating co-movement in daily price changes across commodities. This reflects the tendency of returns to synchronize in response to common information shocks, macroeconomic news, and short-term market sentiment. The dense red structure suggests an ordered regime in which assets respond in an aligned manner. The volatility-based network (right panel) exhibits a mix of positive and negative interactions, revealing greater heterogeneity in risk dynamics. Some commodity groups remain positively connected. In other cases, several blue links indicate divergence in volatility behavior across markets. Volatility captures slower-moving, structural risk channels that are not fully aligned across assets.

It follows that return-based networks emphasize short-term synchronization, whereas volatility-based networks reveal fragmentation and competing risk dynamics.

**Figure 7:**
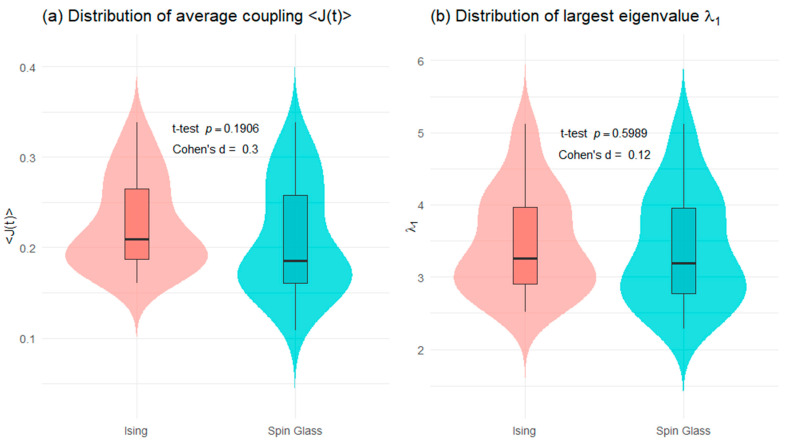
Distributional comparison of aggregate dependence measures: Ising vs. spin glass.

Figure 7 presents a distributional comparison of two aggregate dependence measures $\langle J(t)\rangle$ and the largest eigenvalue $\lambda_{1}\left(t\right)$ computed under the Ising and spin-glass formulations. The violin and box plots show substantial overlap between the two models for both indicators, suggesting very similar empirical behavior. For the average coupling $\langle J(t)\rangle$ (Figure 7a), the mean difference between the Ising and spin-glass models is not statistically significant, as indicated by the two-sample *t*-test (*p* = 0.1906). The corresponding effect size is small (Cohen’s d = 0.30), implying that any difference in average interaction strength across models is modest and economically limited. This result indicates that, at the aggregate level, both formulations encode a comparable degree of overall market connectedness. A similar conclusion emerges for the largest eigenvalue $\lambda_{1}\left(t\right)$ (Figure 7b), which captures the strength of the dominant market mode. The *t*-test yields a high *p*-value (*p* = 0.5989), providing no evidence of a statistically significant difference between the Ising and spin-glass representations. The effect size is negligible (Cohen’s d = 0.12), confirming that both models generate almost identical levels of global synchronization in asset returns.

Table 3 reports statistical tests comparing the distributions of average coupling $\langle J(t)\rangle$ and the largest eigenvalue $\lambda_{1}\left(t\right)$ obtained under the spin glass and Ising representations. The Kolmogorov–Smirnov (KS) test indicates a borderline distributional difference for the average coupling, and accompanied by a small and statistically uncertain effect size. In contrast, the largest eigenvalue exhibits no evidence of distributional differences, as reflected by a very small KS statistic, a high *p*-value, and a negligible effect size. These results suggest that while average coupling may display marginal sensitivity to the modeling framework, the dominant eigenvalue captures robust and model-invariant aggregate dependence dynamics.

**Figure 8:**
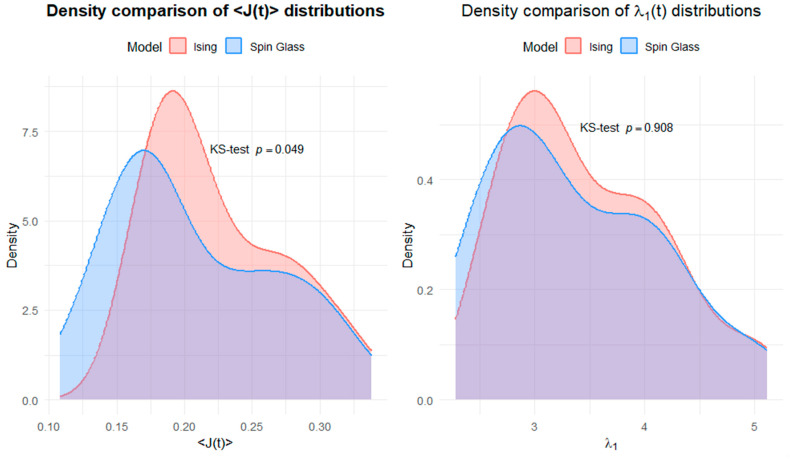
Density comparison of aggregate dependence measures: Ising vs. spin glass.

Figure 8 illustrates how the distributions of the average coupling $\langle J(t)\rangle$ and the largest eigenvalue $\lambda_{1}\left(t\right)$ differ between the Ising and spin-glass models.

For the average coupling $\langle J(t)\rangle$, the left panel reveals moderate differences in distributional shape. The Ising model exhibits a more concentrated density around intermediate coupling values, whereas the spin-glass model shows greater dispersion, with higher probability mass at lower coupling levels and a flatter overall profile. Even though mean differences are limited, the two models differ in terms of variability and tail behavior, reflecting the higher heterogeneity of interaction strengths embedded in the spin-glass framework.

The right panel shows that the distributions of the largest eigenvalue $\lambda_{1}\left(t\right)$ are nearly identical for the Ising and spin-glass models. The density curves overlap across the entire support, with no differences in central tendency or dispersion.

**Figure 9:**
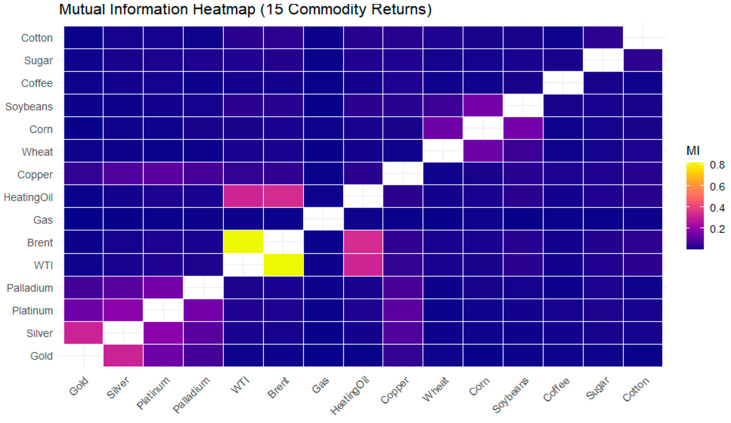
Mutual information heatmap of daily commodity returns.

The *MI* heatmap in Figure 9 provides a detailed representation of nonlinear dependence across the 15 commodity returns. The color scale encodes the strength of pairwise *MI*. Dark blue tones correspond to weak total dependence. Purple and pink shades indicate moderate dependence. Yellow colors represent the strongest interactions. Pronounced yellow and pink regions are observed within the energy commodity group: WTI Crude Oil, Brent Crude Oil, Heating Oil, and Natural Gas. Their strong dependence arises from shared exposure to global energy demand, supply constraints, and geopolitical shocks. The high *MI* values are consistent with the strong linear correlations observed earlier. It follows that dependence within the energy sector is driven by common co-movements.

A similar but less intensive pattern is visible among precious metals (Gold, Silver, Platinum, and Palladium). These interactions are shown in purple to pink shades. The moderate dependence reflects macroeconomic drivers: inflation expectations and financial market uncertainty. The persistence of elevated *MI* suggests that nonlinear interactions may also play a role.

Copper is characterized by dark blue tones in the *MI* heatmap. It indicates weak to moderate dependence with other commodities. Its peripheral role in this dependence structure is highlighted.

Agricultural commodities (Wheat, Corn, and Soybeans) are characterized mainly by dark blue to light purple colors. This indicates weak to moderate total dependence. It follows that price dynamics in agricultural markets are influenced by commodity-specific factors: weather conditions, harvest cycles, and localized supply–demand shocks, rather than by broad systemic drivers.

Soft commodities (Coffee, Sugar, Cotton) are displayed mainly in dark blue, indicating very weak total dependence. This color pattern reflects idiosyncratic price behavior and limited integration with the broader commodity system. These soft commodity markets are driven mainly by local production conditions, weather-related risks, and product-specific demand factors. This pattern of predominantly weak dependence punctuated by sector-specific clusters is consistent with the evidence reported by Jirou et al. [62] for cryptocurrency–financial market networks. They show that dependence links are weak in tranquil periods but undergo structural reorganization and cluster formation during major crisis episodes.

**Figure 10:**
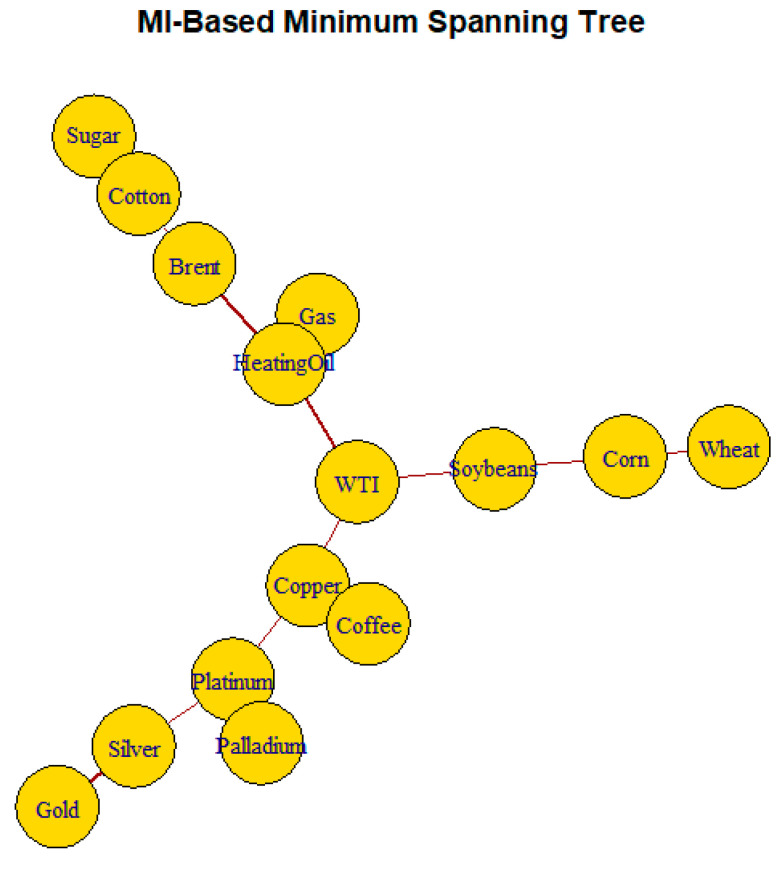
Mutual-information-based minimum spanning tree of commodity returns.

Figure 10 shows the minimum spanning tree (*MST*) constructed from mutual-information-based distances. The resulting structure highlights a clear hierarchical organization across commodity classes. Energy commodities form the core of the network. WTI Crude Oil acts as a central hub linking energy markets to other sectors. Strong connections among WTI, Brent, Heating Oil, and Natural Gas reflect shared exposure to global demand conditions, supply constraints, and geopolitical shocks.

Precious metals appear as a peripheral cluster, indicating moderate internal dependence and weaker integration with the energy core. Agricultural commodities extend as a chain from the energy cluster, suggesting limited indirect dependence. Soft commodities are located at the periphery, confirming their largely idiosyncratic dynamics. Copper occupies an intermediate bridging position, consistent with its role as an industrial metal linked to both energy markets and metals.

The MI-based MST in Figure 10 reveals weak global integration combined with strong sectoral organization. It complements the MI heatmap and supports the view that aggregate dependence measures mask substantial heterogeneity in the interaction structure.

**Figure 11:**
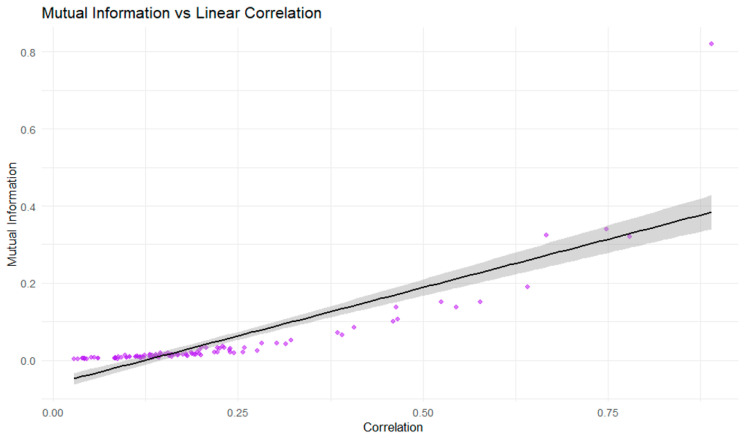
Relationship between mutual information and linear correlation across commodity returns.

Figure 11 illustrates the relationship between pairwise MI and linear correlation for commodity returns. The positive association indicates that higher linear correlations are accompanied by higher levels of MI. This confirms that MI is consistent with correlation when linear dependence is strong.

At the same time, the noticeable dispersion around the fitted line, particularly at low and moderate correlation levels, shows that asset pairs with similar linear correlations can exhibit different MI values. This dispersion indicates that correlation alone does not fully capture the dependence structure. MI reflects additional interaction components not fully explained by linear co-movement.

The MI-based portfolio diversification index is constructed in two steps. First, a rolling average MI is computed across all commodity pairs using discretized returns. For each rolling window, the pairwise MI matrix is estimated using the plug-in estimator. The average of the upper-triangular elements is taken to summarize the system-wide level of dependence at each point in time.

Formally, if ${MI}_{ij}(t)$ denotes the mutual information between commodities i and j in a given rolling window at time t, then the rolling average *MI* is defined as:

(7) $$\bar{MI}\left(t\right)=\frac{2}{N(N-1)}\sum_{i<j}{MI}_{ij}(t)$$

Where *N* is the number of assets and the summation runs over the upper-triangular elements of the MI matrix. The portfolio diversification index (*PDI*) is defined as:

(8) $$PDI\left(t\right)=1-\bar{MI}\left(t\right)$$

By construction, higher *PDI* values correspond to lower average dependence and greater diversification potential, while lower *PDI* values indicate stronger overall dependence and reduced diversification opportunities. The rolling estimation uses a window length of 90 trading days (approximately one quarter), updated every 30 trading days. This overlapping-window design balances two objectives: (i) capturing short- to medium-term changes in dependence structures and (ii) ensuring sufficient observations within each window for stable mutual information estimation.

**Figure 12:**
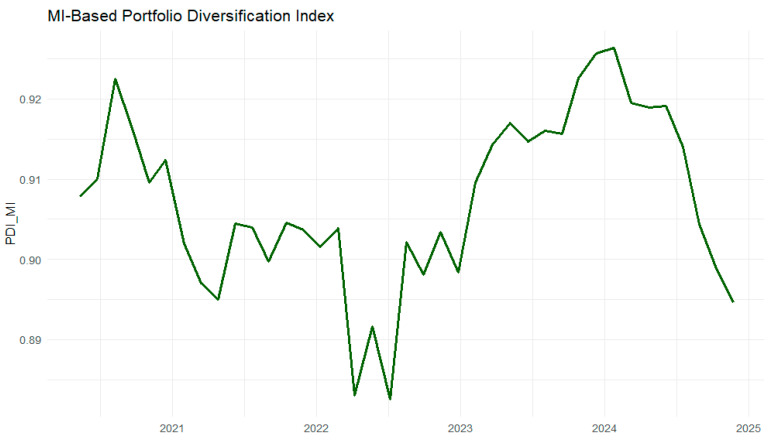
Rolling mutual-information-based portfolio diversification index.

Figure 12 shows that the *MI*-based portfolio *PDI* varies and is closely associated with major global crisis episodes. Pronounced declines in the *PDI* are observed during periods of heightened stress, around the COVID-19 pandemic in 2020 and the Russia–Ukraine war in early 2022. This indicates increased synchronization and reduced diversification across commodity markets. The subsequent recovery during 2023–2024 reflects a partial re-fragmentation of dependence structures, although the decline toward the end of the sample (31 December 2024) suggests that diversification benefits remain sensitive to ongoing global uncertainty.

## 5. Conclusions

This paper compares Ising and spin-glass representations of financial market dependence applied to commodity markets. The empirical results show that both frameworks capture similar dynamics in aggregate synchronization indicators, such as the average coupling strength and the largest eigenvalue. The role of the Ising model as an effective benchmark for market-wide coherence is confirmed. The spin-glass framework reveals richer structural heterogeneity and interaction variability at the network level and during regime transitions.

Even if the synchronization indicators are similar for the two models, the spin-glass framework offers additional information because it retains the sign of interactions. This allows the identification of heterogeneous regimes where some commodities move together and some move in the opposite direction. This feature is suppressed in the Ising model but becomes visible in the network topology and during regime transitions.

The integration of MI and entropy-based diagnostics highlights the importance of nonlinear dependence. At the sectoral level, energy commodities exhibit the strongest and most persistent dependence, forming a dense core in both correlation- and MI-based networks, especially during the 2022 energy shock. Precious metals display moderate but stable interdependence, consistent with their role as safe-haven assets. Industrial metals, represented by Copper, occupy an intermediate and bridging position between energy and other commodity classes. Agricultural commodities and soft commodities show weak and fragmented dependence structures, indicating largely idiosyncratic price dynamics. These patterns explain why aggregate synchronization rises during systemic shocks while diversification opportunities re-emerge in more tranquil periods.

This study has some limitations. A limitation relates to the scope of the dataset. The analysis considers 15 representative commodity markets, which provides a sectoral structure but may not fully reflect the complexity of global financial systems. Extending the analysis to larger cross-asset datasets, including equities, bonds, and currencies, would evaluate whether the observed similarities between Ising and spin glass dynamics remain valid in broader financial environments. Results may be sensitive to methodological choices such as rolling-window length and MI discretization. Interaction structures are treated as quasi-static within each rolling window, limiting the ability to capture very high-frequency structural changes.

The proposed framework has practical implications for financial monitoring. Rising synchronization indicators such as average coupling and maximum eigenvalue show increased market integration. To monitor these indicators in real time may provide early-warning signals of stress propagation among commodity markets. Periods characterized by lower coupling and weaker network connectivity indicate a more fragmented structure in which diversification opportunities increase. Network-based diagnostics can complement traditional risk monitoring tools. The Diebold–Yilmaz connectedness results provide a practical tool for detecting periods of elevated market integration and potential systemic vulnerability.

Future work may extend the framework to larger and more diverse financial systems, incorporate dynamic or agent-based spin-glass models, and explore stochastic extensions to improve early-warning detection of market instability and bubble-like regimes.

## Figures and Tables

**Table 1:** Comparison between Ising and spin glass models.

Property	Ising Model	Spin Glass Model
Interaction ($J_{ij}$)	Deterministic, sign-constrained	Heterogeneous, sign-varying (positive or negative)
Market behavior	Collective alignment (herding)	Frustration, heterogeneity, and competition
Structural organization	Single dominant alignment pattern	Multiple competing interaction patterns
System phase	Ordered vs. disordered regimes	Complex, metastable-like heterogeneous regimes

**Table 2:** Entropy measures.

Shannon Entropy (J)	Rényi Entropy (J)	Tsallis Entropy (J)
4.04	4.16	0.98

**Table 3:** Statistical comparison of spin glass and Ising dependence measures.

Indicator	Test/Measure	Statistic	*p*-Value/CI	Interpretation
Average coupling ⟨*J(t)*⟩	Kolmogorov–Smirnov test	D = 0.308	*p* = 0.049	Borderline distributional difference
	Effect size (Hedges’ g)	g = 0.296	95% CI [−0.153, 0.745]	Small but statistically uncertain effect
Largest eigenvalue *λ_1_(t)*	Kolmogorov–Smirnov test	D = 0.128	*p* = 0.908	Distributions statistically indistinguishable
	Effect size (Hedges’ g)	g = 0.118	95% CI [−0.329, 0.565]	Negligible effect

**Figure A1:**
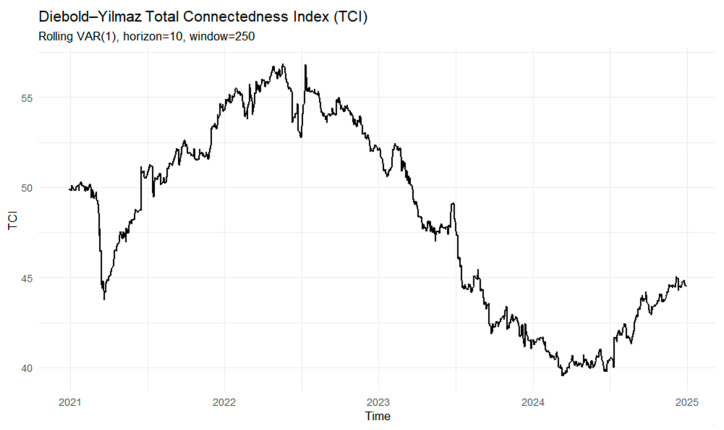
Diebold–Yilmaz Total Connectedness Index (TCI) for commodity markets.

**Figure A2:**
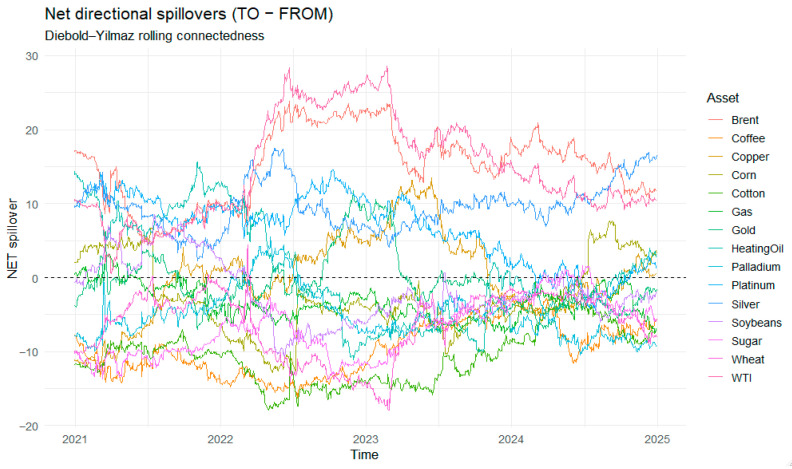
Net directional spillovers in commodity markets (Diebold–Yilmaz framework).

**Figure A3:**
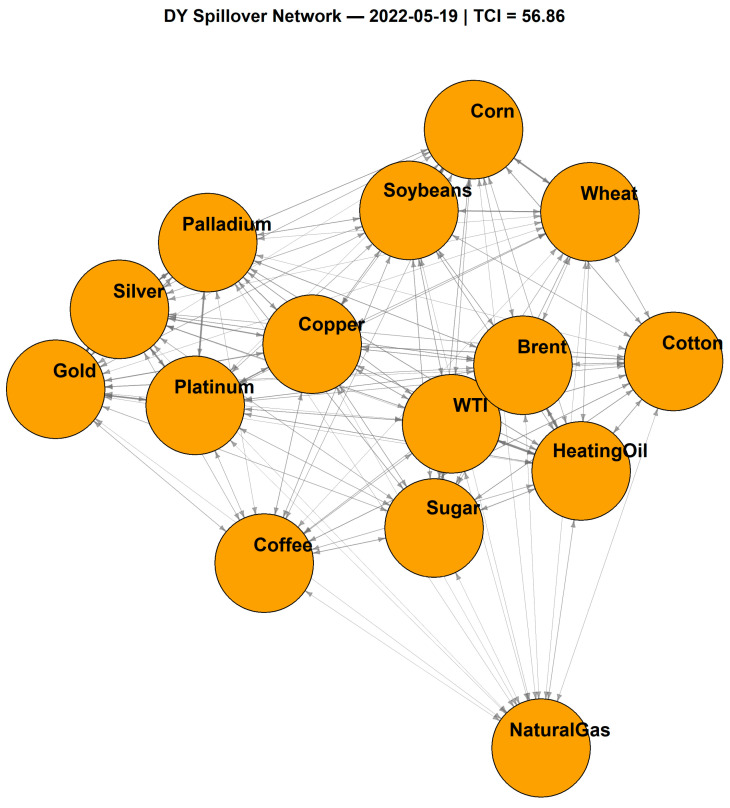
Diebold–Yilmaz spillover network at the peak of systemic connectedness. Note: Directed edges represent net spillover effects derived from the generalized forecast error variance decomposition, where an arrow from asset *i* to asset *j* indicates that shocks in asset *i* transmit to asset *j*.

**Figure A4:**
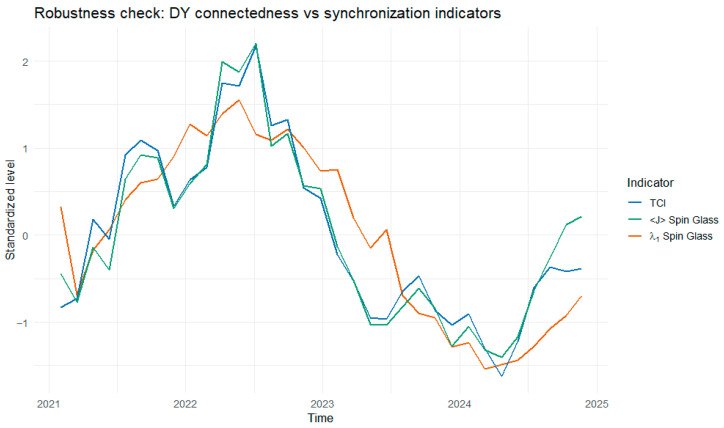
Dynamic relationship between DY connectedness and spin-glass synchronization indicators.

**Figure A5:**
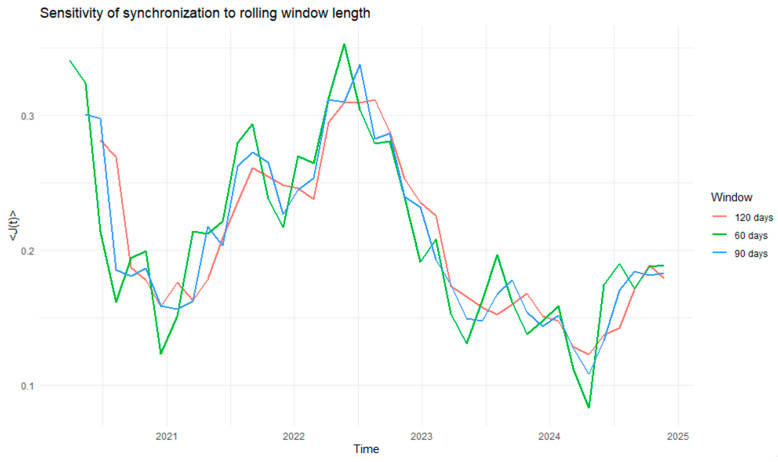
Sensitivity of the average coupling $\langle J(t)\rangle$ to rolling window length.

**Figure A6:**
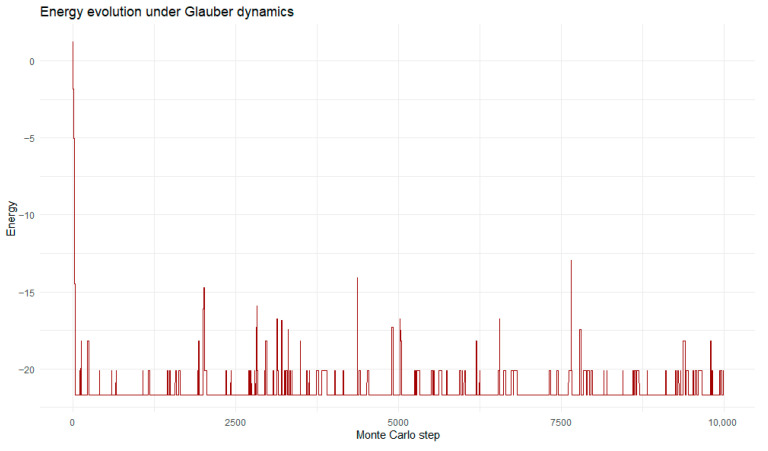
Energy evolution of the commodity interaction system under Glauber dynamics.

**Figure A7:**
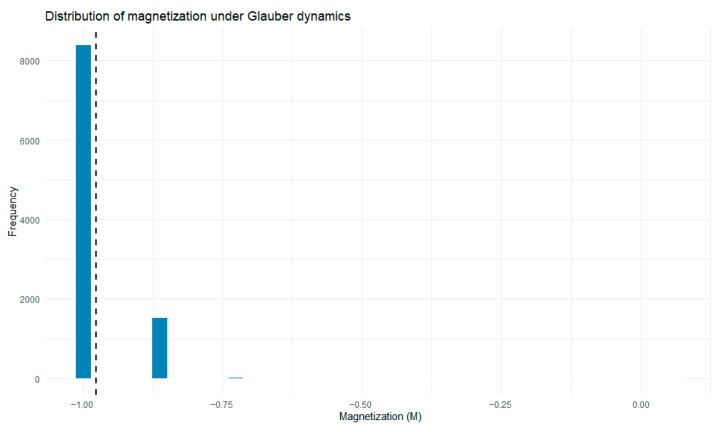
Distribution of magnetization of the commodity interaction system under Glauber dynamics. Note: The dashed vertical line denotes the mean magnetization, providing a reference for the central tendency of the system under Glauber dynamics.

## Data Availability

Data is available upon reasonable request.

## Appendix A. Diebold–Yilmaz Connectedness Analysis

To complement the dependence measures used in the main analysis, we include a robustness analysis based on the connectedness framework developed by Diebold and Yilmaz [63,64]. While the coupling matrix J constructed from correlations and mutual information captures the strengths of statistical dependence between assets, these measures are symmetric and therefore do not identify the direction of information transmission across markets. In financial systems shocks often propagate asymmetrically across assets and sectors. To address this limitation, we estimate directional spillovers using the methodology proposed by Diebold and Yilmaz [63,64], which measures connectedness using forecast error variance decompositions derived from vector autoregressive (VAR) models. Baruník and Křehlík (2018) [65] extend the Diebold–Yilmaz connectedness framework by decomposing spillovers into frequency components, allowing the distinction between short-term and long-term sources of systemic risk transmission. The forecast error variance of each variable is decomposed into contributions originating from shocks to all other variables in the system. This allows the construction of measures of total, directional, and net spillovers across markets. In particular, the Total Connectedness Index (TCI) summarizes the proportion of forecast error variance that is explained by cross-market spillovers rather than by idiosyncratic shocks. Higher values of the index indicate stronger systemic interdependence and more intense information transmission across markets.

Figure A1 reports the evolution of the TCI for the commodity system over the sample period using a rolling VAR(1) model with a forecast horizon of ten days and a window size of 250 trading days. The results reveal time variation in systemic connectedness. In early 2021, TCI fluctuates around 50%, indicating a moderately integrated commodity system. Connectedness rises sharply in 2022, exceeding 56–57%, which represents the highest level in the sample. This increase coincides with major geopolitical shocks, particularly the Russia–Ukraine conflict, and reflects stronger cross-market spillovers.

Then TCI declines during 2023 and reaches its lowest levels in 2024, around 40%, suggesting a more fragmented market structure. Toward the end of 2024, TCI increases to about 44–45%. Periods of stronger synchronization in the Ising and spin-glass frameworks correspond to higher directional spillovers in the DY connectedness analysis.

Figure A2 reports the net directional spillovers (NET = TO − FROM) obtained from the Diebold–Yilmaz connectedness framework. Positive values indicate commodities that act as net transmitters of shocks, while negative values indicate net receivers. The results show that energy commodities, particularly WTI, Brent, and Heating Oil frequently appear as net transmitters, especially during 2022–2023, when global energy markets experienced geopolitical and supply shocks [66]. Several agricultural commodities, such as Wheat, Soybeans and Corn, more often behave as net receivers, absorbing shocks originating from other sectors. Precious metals, including Gold and Silver, have a more balanced role in time, alternating between transmitting and receiving shocks depending on market conditions. These patterns are consistent with the evolution of TCI and with synchronization effects identified in the spin glass and Ising models. Periods of higher systemic interaction coincide with stronger spillovers originating mainly from the energy sector.

The average TCI of about 48% in Table A1 indicates that about half of the forecast error variance in commodity returns is explained by cross-market spillovers. The range from 39.55% to 56.86% shows that directional connectedness varies in time, increasing during periods of stronger market synchronization.

**Table A1:** Summary statistics of the Diebold–Yilmaz TCI.

Statistic	Value
Mean TCI	48.25
Median TCI	48.82
Minimum TCI	39.55
Maximum TCI	56.86

Note: The TCI is computed using a rolling VAR(1) model with a forecast horizon of 10 days and a window size of 250 trading days.

Figure A3 presents the spillover network on 19 May 2022, the date at which TCI reaches its maximum values. The snapshot illustrates the structure of directional spillovers during the period of strongest systemic interaction in the commodity market. Edge thickness is proportional to the estimated spillover intensity obtained from the variance decomposition. Thicker connections identify the dominant channels of shock propagation in the commodity network. The network suggests that energy commodities (e.g., Brent, WTI, and Natural Gas) play a central role in the transmission of shocks, while agricultural commodities such as Wheat, Corn, and Soybeans appear more influenced by incoming spillovers, although the dense connectivity indicates that interactions are highly interconnected across all asset classes.

Figure A4 compares the evolution of the Diebold–Yilmaz TCI with the synchronization indicators derived from the spin glass framework, namely $\langle J(t)\rangle$ and the largest eigenvalue $\lambda_{1}$. By standardization, all three indicators are compared on the same scale. Figure A4 shows a clear co-movement between the three measures. Periods of higher systemic connectedness, especially during 2022, coincide with rises in both $\langle J(t)\rangle$ and $\lambda_{1}$, indicating strong market synchronization. The decline noticed during 2023–2024 is reflected among all indicators, suggesting a reduction in directional spillovers and couplings in the commodity systems. The similar dynamics support the interpretation that higher synchronization in the spin glass representation is associated with stronger directional connectedness captured by the Diebold–Yilmaz framework.

## Appendix B. Sensitivity Analysis and Robustness Checks

Figure A5 illustrates the evolution of the average coupling indicator $\langle J(t)\rangle$ computed using alternative rolling windows of 60, 90, and 120 days. The three series follow similar dynamics throughout the studied period, indicating that the estimated synchronization patterns are not sensitive to the specific window choice. The increase in coupling during 2022 and the subsequent decline during 2023–2024 appear consistently for all specifications.

In Table A2, the correlations between the synchronization indicators computed with different rolling-window lengths are all above 0.80. This strong association indicates that the dynamics of the average coupling $\langle J(t)\rangle$ are robust to reasonable changes in the window specification. This supports the stability of the main results.

**Table A2:** Correlation of the average coupling $\langle J(t)\rangle$ across alternative rolling windows.

Window Length	60 Days	90 Days	120 Days
60 days	1.000	0.913	0.833
90 days	0.913	1.000	0.934
120 days	0.833	0.934	1.000

To evaluate the sensitivity of the dependence estimates to discretization choices, *MI* matrices were computed using both equal-width and equal-frequency binning schemes. The resulting matrices are highly correlated (=0.85), indicating that the dependence structure remains stable. Therefore, the interaction network used in the analysis is not driven by a particular discretization method.

## Appendix C. Glauber Dynamics Simulation

To complement the static interaction analysis from above, we introduce a dynamic framework based on Glauber dynamics [44], which is frequently used to simulate the time evolution of the Ising model. The approach is based on a probabilistic mechanism through which interacting units update their states in time and move to equilibrium.

In the Ising representation, each element $s_{i}\in \{-1,1\}$. The interaction between elements is governed by the coupling matrix $J_{ij}$. The Hamiltonian of the system is [13]:

(A1) $$H\left(s\right)=-\sum_{i<j}J_{ij}s_{i}s_{j}$$

where positive couplings represent alignment, while negative ones generate frustration. Using an Ising representation of spins, with random couplings $J_{ij}$, the system becomes a spin-glass model [67].

Glauber dynamics introduces updates to the system. At each step an element is selected and its state is flipped with probability [44]:

(A2) $$P\left(s_{i}\to s_{j}\right)=\frac{1}{1+exp(\frac{∆E_{i}}{T})}$$

$∆E_{i}$ represents the change in energy and *T* is a temperature parameter controlling randomness. The change in energy associated with flipping spin $s_{i}$ is [44]:

(A3) $$∆E_{i}=2s_{i}\sum_{j}J_{ij}s_{j}$$

In our empirical setting, the simulation is performed for *N* = 15 commodity markets using the following steps:

Step 1. Initialize spins randomly $s_{i}\in \{-1,1\}$.

Step 2. Select a random asset *i*.

Step 3. Compute the local field: $h_{i}=\sum_{j}J_{ij}s_{j}$.

Step 4. Compute the energy change using Equation (A3).

Step 5. Flip the spin with probability given in Equation (A2).

Step 6. Repeat for 10,000 Monte Carlo steps.

After this, we compute the system magnetization defined as [7]:

(A4) $$M\left(t\right)=\frac{1}{N}\sum_{i=1}^{N}s_{i}$$

*M* measures the overall alignment of the system. High absolute values of *M* indicate strong collective behavior or herding. Near 0 values correspond to weakly correlated dynamics. In financial terms, this quantity has been used to represent market sentiment, aggregate demand or collective price movements. Market participants are interpreted as spins, while spin interactions generate market behavior such as bubbles, crashes or volatility clustering [7].

Figure A6 shows the evolution of the system energy during the Monte Carlo simulation driven by the empirical coupling $J_{ij}$. Starting from a random configuration, the energy decreases and gets stable around lower values. This means that the system moves to a more stable configuration determined by the interaction structure among the commodities.

The histogram in Figure A7 shows that the concentration of variables around M≈−1 indicates that most commodities tend to align in the same direction. This reflects a high level of collective behavior generated by the empirical interaction matrix. The dashed vertical line represents the average magnetization, pointing to the tendency of the system toward a coordinated regime.
